# Home spirometry telemonitoring in pediatric patients with asthma: a mixed study

**DOI:** 10.3389/fped.2025.1554921

**Published:** 2025-05-14

**Authors:** Dingrong Fan, Ao Shen, Qianqian Wang, Lu Peng, Ruixue Xia, Hengyu Zhou

**Affiliations:** ^1^ School of Nursing, Chongqing Medical University, Chongqing, China; ^2^ Department of Pediatrics, the Second Affiliated Hospital of Chongqing Medical University, Chongqing, China

**Keywords:** home spirometry, telemonitoring, pediatric, asthma, feasibility, practicality

## Abstract

**Background:**

To evaluate the feasibility and practicality of home spirometry telemonitoring for pediatric patients with asthma, including both motivators and barriers, as well as the requirements for effective implementation.

**Methods:**

This single-arm, prospective study involved three phases: outpatient spirometry examination, home spirometry telemonitoring, and semi-structured interviews. A total of 110 children aged 5–12 years, who required spirometry monitoring at the pediatric outpatient clinic of the Second Affiliated Hospital of Chongqing Medical University, were enrolled. The PF286 (referred to as PF286), a home spirometry telemonitoring device was used for this study. Upon enrollment, each child initially underwent spirometry with a clinical-grade spirometer (Jaeger). Subsequently, they were tested using the PF286 under both supervised and unsupervised settings. To assess the consistency between PF286 and the clinical spirometer, we employed Pearson correlation coefficients. Children diagnosed with mild-to-moderate asthma, based on specified tests, participated in a four-week home spirometry telemonitoring program. After the telemonitoring period, semi-structured interviews were conducted with children, their guardians, and healthcare professionals to evaluate their experiences and identify the motivators and barriers in integrating home spirometry telemonitoring into clinical care. Spirometry data were collected using the PF286.

**Results:**

The findings suggest that the PF286 is suitable for home spirometry telemonitoring in children, with unsupervised use yielding reliable data for clinical application. Semi-structured interviews with eight groups of children and their guardians, five nurses, and four physicians identified five key themes: “benefits of telemonitoring”, “data accuracy and reliability”, “barriers”, “emotions”, and “ expectations”.

**Conclusion:**

The study concludes that home spirometry telemonitoring is feasible and acceptable for pediatric asthma management. However, several challenges, such as cost, insurance coverage, data security, health education, and healthcare workload, need to be addressed prior to its widespread implementation. Future research should focus on leveraging artificial intelligence for early disease detection, treatment guidance, and improving the quality of life for pediatric asthma patients.

## 1 Introduction

Asthma is a highly prevalent chronic condition among children with global prevalence estimates projected to exceed 400 million by 2025, and affects approximately 10% of the pediatric population worldwide (1–3). Characterized by recurrent wheezing, airflow limitation, and obstructive ventilatory dysfunction, asthma is linked to significantly functional impairments, reduced physical activity, school absenteeism, and psychological distress in affected children (4). Current clinical guidelines recommend spirometry for the diagnosis and assessment of asthma control in pediatric patients (5). However, outpatient spirometry faces challenges related to accessibility and continuity of care (6). The COVID-19 pandemic has accelerated the adoption of home-based spirometry telemonitoring, which has shown potential in enhancing self-management strategies for asthma among children (7–11).

The efficacy of home spirometry telemonitoring, despite its widespread adoption, remains a continues issue within the medical community (12). A recent comprehensive review revealed that only three out of ten devices from various manufacturers met the requisite technical standards considered acceptable (13). Consequently, any new portable spirometer must undergo rigorous validation prior to its implemented in clinical practice. Moreover, a consensus on the effectiveness of home spirometry telemonitoring has yet to be reached, with a lack of definitive studies demonstrating equivalence between unsupervised home telemonitoring and supervised clinical spirometry data (14).

Current research on home spirometry telemonitoring for asthmatic school-aged children has predominantly been conducted in Western countries, with a notable absence of studies from Eastern regions (15–17). Cultural differences between Eastern and Western parenting paradigms may significantly impact children's self-management abilities (18). Furthermore, there is a notable lack of research investigating how Chinese families and healthcare providers perceive and utilize this emerging technology in asthma management. This mixed-method study aims to provide new insights into the effects of home-based remote spirometry telemonitoring on pediatric asthma care. To achieve this goal, the concordance between the remote smart spirometer PF286 and clinical assessments was rigorously examined and validated. Additionally, the feasibility and adherence were evaluated to unsupervised home spirometry telemonitoring. We also conducted semi-structured interviews with Chinese families and healthcare professionals to understand their perspectives on this. This study offers compelling evidence that highlights the efficacy of home spirometry telemonitoring in enhancing self-management among Chinese children with asthma.

## 2 Methodology

### 2.1 Experiments

We conducted a single-arm, prospective mixed-method study to evaluate the YouBreathe home spirometry telemonitoring device, PF286, and its associated telemonitoring platform (hereinafter referred to as PF286), both provided by Zhejiang Yiliankang Medical Science and Technology Co., Ltd. We declare no conflicts of interest involving the device manufacturer, members of the ethics committee, guardians of the participants, or in relation to the disclosure of the findings. Ethical approval for this research was granted by Chongqing Medical University (Ethical Approval No. 2023095). Prior to enrollment, informed consent was obtained from all participating children through their guardians. This study was organized into three distinct phases, each aligned with specific research objectives.

### 2.2 Device

PF286 is a handheld home spirometer designed to measure commonly used spirometry parameters, including forced vital capacity (FVC), forced expiratory volume in one second (FEV1), percentage of FEV1/FVC in one second (FEV1/FVC), peak expiratory flow rate (PEF), and forced expiratory flow at 25%–75% of FVC (FEF25-75). Before using the device, the child's information is linked to both the public WeChat terminal and the platform terminal. The device initiates with an automatic zero calibration, and the children are guided to exhale into it following voice prompts. The built-in algorithm conducts intelligent quality control of the results and visual displays. The device uses color coding to indicate the severity of the measurements, with green indicating that no intervention is needed, while yellow or red indicates that the patient's PEF is below 80% of the predicted value, suggesting the need for medical consultation and treatment.

### 2.3 Phase 1: outpatient spirometry tests

#### 2.3.1 Population

From September 2023 to August 2024, pediatric patients requiring spirometry assessments were referred to the pediatric outpatient department of the Second Affiliated Hospital of Chongqing Medical University for the diagnostic procedure.

#### 2.3.2 Inclusion criteria

Children aged 5–12 who demonstrated the ability to complete acceptable spirometry during routine clinical visits.

#### 2.3.3 Exclusion criteria

Patients were excluded from spirometry testing for the following contraindications: (1) Pneumothorax or alveolar abnormalities; (2) Significant cardiac arrhythmias or other medical conditions; (3) Perforated eardrums caused by otitis media; (4) Hemoptysis within the last month; (5) Active tuberculosis or ongoing anti-tuberculosis therapy; (6) Respiratory infections; (7) Recent chest, abdominal, or eye surgeries (within 1–3 months); (8) Seizures requiring pharmacological intervention; (9) Inguinal or umbilical hernias at risk of incarceration; (10) Inability to cooperate with spirometry testing (e.g., cognitive impairments).

#### 2.3.4 Data collection

Upon enrollment, children were initially assessed using a large, clinical-grade spirometer (Jaeger) to establish a baseline. Following a mandatory 10-minute rest period, both the children and their guardians received standardized training on the proper operation of the PF286 instrument. Subsequently, under the direct supervision of a qualified technician, the children completed the PF286 test. After another required 10-minute rest, they were then asked to complete the PF286 test unsupervised—either independently or with their guardians assistance. Throughout these process, measurement techniques and data quality control were rigorously maintained in accordance with current guidelines established by the European Respiratory Society (ERS) and American Thoracic Society (ATS) (19).

### 2.4 Phase 2: home spirometry telemonitoring

#### 2.4.1 Participant recruitment

According to the standardized diagnosis and treatment recommendations for pediatric asthma in China and the Childhood Asthma Control Test (C-ACT) (20, 21), families were invited to participate in a 4-week home spirometry program following the diagnosis of mild to moderate asthma in their children after the initial examination.

#### 2.4.2 Data collection

During weeks 1–2 after enrollment, children underwent telemonitoring with direct supervision by technicians through Tencent Conference. In weeks 3–4, the children were monitored unsupervised, either with their parents or independently. To ensure compliance, technicians reviewed daily test results at 21:00 and contacted children who missed measurements for three consecutive days to determine the reasons for non- adherence. Throughout the study period, children's asthma control was monitored using the C-ACT questionnaire to prevent adverse events. In the event of illness during the telemonitoring, this would be immediately suspended, and the study team assessed the severity of the adverse event and its potential correlation with remote home pulmonary function telemonitoring. Children who experienced injuries or complications resulting from the treatment would receive free care at the Second Affiliated Hospital of Chongqing Medical University, and any serious adverse events were reported to the Ethics Committee within 48 h.

### 2.5 Phase 3: semi-structured interviews

#### 2.5.1 Participant recruitment

After the home spirometry telemonitoring, the child's guardians were contacted to arrange a follow-up appointment at our clinic. Additionally, children, their guardians, and healthcare professionals involved in the consultation were invited to participate in a semi-structured interview gather insights into their experiences and perceptions.

#### 2.5.2 Interview framework

The outline for the interviews was initially crafted through a comprehensive literature review and the utilization of the STAR tool from the Behavioral Event Interviewing Method (BEIM) (22). The interview guide was subsequently refined through expert consultation and pilot testing with three families of children with asthma, as well as three healthcare professionals.

For the parent interviews, we focused on key topics, including the ability of home telemonitoring to facilitate earlier detection of changes in their child's lung function, the lifestyle implications of home monitoring compared to traditional hospital visits, and extent to which this approach has empowered them in managing their child's condition. Additional 15 questions were included in interviews.

The healthcare professional interviews focused on issues encountered while using the technology, their views on the effect of remote lung function monitoring on follow-up schedules and check-up frequency for pediatric respiratory patients, its potential to expedite the detection of changes in patients’ conditions and assess treatment efficacy, and its potential to reduce the workload of hospitals and physicians. Another 16 questions were included in the interviews.

#### 2.5.3 Data collection

By employing a phenomenological approach in the qualitative research, semi-structured in-depth interviews were conducted with family groups engaged in home monitoring and healthcare professionals involved in the consultations. Prior to the interviews, the interviewer introduced themselves and provided a comprehensive explanation of the purpose, methodology, components, and time required for participation. Participants were assured of confidentiality regarding their interview data, and informed consent was obtained prior to conducting face-to-face interviews, which were audio-recorded for accuracy. Each interview, lasting approximately 20–60 min, was conducted in a designated clinic room to ensure privacy and comfort. During the interview, the interviewer attentively listened, observeed, and meticulously recorded the respondent's nonverbal behaviors while probing deeper into specific issues as dictated by the context. After each interview was recorded and transcribed into a written format, the transcript was returned to the respondent for validation, ensuring data accuracy. The process of interviewing continued until data saturation was reached.

### 2.6 Statistical analysis

#### 2.6.1 Quantitative analysis

The data analysis was conducted using SPSS 22.0, with descriptive statistics presented as mean and standard deviation (*x* *±* *s*). To assess changes within the same group over time, paired *t*-tests were employed for pre- and post-comparisons. The ICC consistency along with Bland-Altman plots were used to assess variable relationships. The coefficient of variation was used to evaluate the degree of indicator dispersion. Statistical significance was set at the conventional threshold of *P* < 0.05. Compliance was defined as the number of actual measurements divided by the number of requested measurements, multiplied by 100%.

#### 2.6.2 Qualitative analysis

The qualitative analysis of the interview content was analyzed using Colaizzi's 7-step method (22) with Nvivo 11.0 for data management and support analysis. Two researchers independently conducted the thematic coding. Subsequently, the emergent themes were discussed by the entire research team to achieve consensus.

## 3 Results

### 3.1 Baselinea patient characteristics

A total of 110 children were recruited during the outpatient pulmonary function test phase, 47.27% were female and 52.73% male, the average age was 7.21 ± 2.54 years, with a BMI of 15.56 ± 2.91 kg/m². 52.73% of families had an annual income <100,000 yuan. Parental education levels were predominantly associate degrees (39.09%) and bachelor's degrees (34.55%). 39 children were enrolled in the group of telemonitoring at home and their general information is as follows:the mean age was 7.21 years [standard deviation (SD) ± 1.44], with 25 males (64.1%) and 14 females (35.9%). The mean height was 126.04 cm (SD ± 10.49), and the mean weight was 27.22 kg (SD ± 8.45). Family income distribution showed that 27 families (69.23%) had an annual income of CNY 80,000–150,000, while 12 families (30.77%) earned CNY 150,000–300,000. All participants (39 cases, 100%) were assessed as having “well-controlled” disease status.

### 3.2 Consistency analysis between jaeger, outpatient PF286 supervised and outpatient PF286 unsupervised

The Intraclass Correlation Coefficient (ICC) consistency test was performed to assess the agreement among Jaeger, Outpatient PF286 supervised, and Outpatient PF286 unsupervised. The results demonstrated excellent consistency for FVC% prediction (ICC = 0.912, 95% CI: 0.882–0.936, *P* < 0.001), FEV1% prediction (ICC = 0.946, 95% CI: 0.927–0.961, *P* < 0.001), and PEF% prediction (ICC = 0.974, 95% CI: 0.964–0.981, *P* < 0.001) across all three groups. Detailed results are presented in Table 1.

**Table 1:** Consistency analysis between jaeger, outpatient PF286 supervised and outpatient PF286 unsupervised.

Parameters	Jaeger	Outpatient PF286 supervised	Outpatient PF286 unsupervised	ICC	95%CI	*P*-value
FVC%	105.07 ± 11.34	104.42 ± 12.24	104.01 ± 15	0.912	0.882–0.936	<0.001
FEV1%	106.91 ± 11.56	105.85 ± 15.65	105.94 ± 15.83	0.946	0.927–0.961	<0.001
PEF%	96.7 ± 15.05	95.75 ± 17.84	96.14 ± 18.17	0.974	0.964–0.981	<0.001

### 3.3 Consistency analysis between jaeger, PF286 supervised home telemonitoring, PF286 unsupervised home telemonitoring

Consistency analysis of pulmonary function parameters among Jaeger, 14-day home-based PF286 supervised, and 14-day home-based PF286 unsupervised revealed strong agreement across all groups. The intraclass correlation coefficients (ICCs) were 0.947 (95% CI: 0.928–0.962, *P* < 0.001) for FVC%, 0.949 (95% CI: 0.931–0.963, *P* < 0.001) for FEV1%, and 0.967 (95% CI: 0.967–0.983, *P* < 0.001) for PEF%, indicating excellent inter-group consistency. Complete data are shown in Table 2.

**Table 2:** Consistency analysis between jaeger, PF286 supervised home telemonitoring, PF286 unsupervised home telemonitoring.

Parameters	Jaeger	PF286 supervised home telemonitoring	PF286 unsupervised home telemonitoring	ICC	95%CI	*P*-value
FVC%	105.07 ± 11.34	103.8 ± 15.45	104.11 ± 15.28	0.947	0.928–0.962	<0.001
FEV1%	106.91 ± 11.56	105.56 ± 15.8	106.11 ± 15.63	0.949	0.931–0.963	<0.001
PEF%	96.70 ± 15.05	95.52 ± 17.92	95.25 ± 17.46	0.976	0.967–0.983	<0.001

### 3.4 Compliance of home spirometry telemonitoring

Participant compliance rates demonstrated a progressive weekly decline: 92.67% in Week 1 (monitoring attempts = 253), decreasing to 87.55% in Week 2 (*n* = 239), 84.25% in Week 3 (*n* = 230), and a significant reduction to 69.23% in Week 4 (*n* = 189). Supervised home monitoring exhibited the highest compliance rate (90.11%, *n* = 492), significantly outperforming unsupervised modalities (76.74%, *n* = 419; *χ*² = 34.2, *P* < 0.001). The overall compliance rate across all monitoring types was 83.42%.

### 3.5 Semi-structured interviews

This qualitative study involved two participant groups: (1) Family Group: 8 families of asthmatic children, including 7 mothers (87.5%) and 1 father (12.5%) as interviewees. The patients were aged 5–10 years (median 6 years), with Childhood Asthma Control Test (C-ACT) scores ranging from 19 to 25 (mean 21.8 ± 2.1), all meeting the asthma control threshold (>19 points). (2) Healthcare Professional Group: 8 respiratory department staff, comprising 5 bachelor's degree holders (62.5%), 2 master's degree holders (25%), and 1 medical doctorate holder (12.5%). Their titles included 3 chief physicians, 1 associate chief physician, 1 attending physician, and 4 nursing staff (registered nurses/charge nurses). Professional experience spanned 5–24 years (nursing staff averaged 7.2 years; physician team averaged 17.3 years), with ages ranging from 29 to 52 years, forming a multi-tiered team combining senior and junior members (37.5% ≤35 years old; 37.5% having ≥20 years of experience).These interviews yielded five key themes related to home spirometry telemonitoring were identified: ‘Benefits of telemonitoring’, ‘Data Accuracy and Reliability’, ‘Barriers’, ‘Emotional Impact’, and ‘Future Expectations’.

#### 3.5.1 Theme 1: benefits of monitoring

Home spirometry telemonitoring represents a transformative approach in the management of pediatric respiratory diseases, significantly enhancing the efficiency and convenience of healthcare delivery. It fosters closer collaboration and communication between pediatric patients and medical professionals, thereby positively impacting family health management and overall quality of life.

**Table:** 

Theme 1	Sub-theme	Citation explanation
Benefits of monitoring	Convenience and Flexibility	N6: “This home monitoring method really saves us a ton of time and effort compared to regular hospital check-ups, plus it's super convenient for dealing with minor symptoms. I don't even have to go to the hospital anymore.”!
	Timeliness and Continuity	N17: “Telemonitoring gives us a great chance to spot any changes in the patient's condition quickly. Parents can keep an eye on things from home, and if anything unusual pops up, the medical team can alert us right away. This way, we can tweak the treatment plan or set up any necessary medical help promptly to prevent the patient's condition from getting worse”.
	Enhanced Disease Management Autonomy	N8: “After using the tool, I was able to make some independent calls about how my child is doing.”.
	Avoiding Cross-infection	N5: “A few months back, there was a huge flu outbreak, so we kept an eye on ourselves at home to avoid spreading it and keep our kids safe”.

#### 3.5.2 Theme 2: data accuracy and reliability

In the home environment, proficiently trained users can obtain test results that closely mirror clinical standards. Although occasional errors may occur due to improper handling, these anomalies do not substantially compromise the overall reliability of the data and can be addressed through standardized usage protocols and comprehensive training. Consequently, these minor discrepancies are unlikely to have a substantial impact on clinical assessments and subsequent treatment decisions.

**Table:** 

Theme 2	Sub-theme	Citation explanation
Data accuracy and reliability	Accuracy	N8: “Prior to measuring my child's cough with an instrument, I observed that the recorded value was slightly lower than usual; however, it indicated a green status, leading me to believe it still accurately represented my child's condition”.
	Reliability	N4: “Before I got started, I was worried about the data quality and thought it wouldn't be fair to compare these numbers with clinical exam results. But looking at the monitoring curve, it turns out that most of the tests actually met the quality control standards”.

#### 3.5.3 Theme 3: barriers

Non-professionals may encounter challenges in comprehending the specific indicators associated with spirometry monitoring, which necessitates expert guidance and interpretation to assist parents and patients in accurately interpreting the data. Furthermore, continuous public education, enhanced policies and regulations, along with managerial support will be pivotal in advancing the development of remote pulmonary function monitoring technology.

**Table:** 

Theme 3	Sub-theme	Citation explanation
Barriers	Insufficient patient education	N12: “I'm still having a bit of trouble with these numbers, so I can only tell them apart by color. Red means there's an issue, and green means everything's good”.
	User acceptance varies	N10: “This tech is still pretty new, and using it at home makes me feel a bit uneasy and unsure”.
	Policy	N15: “The costs for getting this tool aren't covered by insurance, and we haven't accounted for the extra labor costs that came up while implementing this technology”.
	Workload	N9: “Each measurement only takes a short amount of time, but it does add an extra layer of planning to family life, and kids don't always adjust to it easily”. N6: “I conduct daily assessments to monitor the children's health status, most of this work is completed during breaks and after work, which adds a certain amount of workload to us”.
	Information Security Management	N5: “I'm a bit worried about data security. Where's the data for kids being uploaded to? Who can access it? And will it be used for anything else?”.

#### 3.5.4 Theme 4: emotions

The emotional responses of patients and their parents during the family monitoring program are varied; however, feelings of insecurity and concern persist, necessitating a gradual adaptation process for both parents and medical staff in clinical practice.

**Table:** 

Theme 4	Sub-theme	Citation explanation
Emotions	Positive	N14: “Right now, the relationship between doctors and patients is moving more towards a shared decision-making approach. As healthcare professionals, we need to understand that both the child and their parents are key partners in making decisions, and that family monitoring data should be included in our treatment plans”.
	Anxiety	N13: “I'm honestly a little concerned that we depend too much on online data interpretation and overlook physical exams and important lab tests. What do we do if there's a disagreement and no clear laws or rules to follow?”.
	Deficiency in self-assurance.	N12: I monitor my child's progress at home myself, and even though all the numbers look good and the doctor says not to worry, I still can't shake this feeling of insecurity. I'm just not confident in what I know and I'm scared of missing something about my child's condition”.
	Equanimity	N6: “Well keep an eye on ourselves like the doctor said, and you can't go wrong by following their advice. I'll stick to what the doctor told me and reach out in the group if I run into any issues. No need to stress about it”.

#### 3.5.5 Theme 5: expectation

A consensus among the majority of medical professionals and family members of patients express a favorable outlook on remote pulmonary function monitoring, positing that with the ongoing advancements in technology, this approach is poised to become a crucial component in the management of pediatric respiratory diseases. Furthermore, the convergence of artificial intelligence and advanced big data analytics is seen as significant promise for enhancing quality control and analysis within home monitoring of childhood asthma; the integration of large datasets and sophisticated algorithms is expected to catalyze a transformation in disease management paradigms.

**Table:** 

Theme 5	Sub-theme	Citation explanation
Expectation	Optimism	“N14: I think with technology moving forward, it's becoming a trend, and honestly, it's smart to be ready for it ahead of time”.
	Intelligentization	N7:“Even though I can use colors to get a general idea of a child's health, I'm really looking for more detailed insights. If AI could automatically analyze this data and turn it into easy-to-understand words or specific advice, along with tips on things like weather, diet, and exercise, that would be awesome!”.
	Forecasting diseases	N16:“The establishment of disease prediction models derived from the analysis of these monitoring data would significantly enhance our physician's ability to make more accurate diagnoses and formulate tailored treatment plans”.
	Dissemination of scientific knowledge	N8: “I really hope the system can give me personalized educational videos and articles that fit my child's needs. For instance, I'd love to know how to use storage containers or if these medications will impact my child's vaccinations, and so on. There are just so many resources and videos out there, and it takes me forever to find what I'm looking for. If the system could offer tailored recommendations, it would save me a ton of time and effort”.
	Policy support	N15:“The government and healthcare organizations really need to step up their game by offering more technical support and financial backing, like including this in the health insurance system, setting standard prices, and improving related policies and regulations. That's the only way we can ensure that technology keeps getting better in the future”.

## 4 Discussion

The strength of this study lies in its mixed—methods design, which combines quantitative device validation with qualitative interviews. The study not only rigorously validated the consistency of the PF286 device with A clinical—grade spirometer (Jaeger), proving its reliability and effectiveness under both supervised and unsupervised conditions, filling a research gap in this region, it also offers practical suggestions for future promotion. Through semi—structured interviews, it thoroughly explored the experiences, perceptions, facilitators, and barriers of children, guardians, and healthcare professionals regarding remote monitoring technology. Additionally, it examined the acceptance and utilization of emerging technologies by Chinese families and healthcare providers, which is crucial for promoting suitable technologies in developing countries and informing relevant policies.

In this study, the consistency of the home spirometry device PF286 with clinical Jaeger spirometry was validated. There was strong agreement between the PF286 and clinical Jaeger results in children aged ≥5 years with successful spirometry (ICC > 0.9). Similar results were observed during home telemonitoring (ICC > 0.9). In the interviews, both parents and healthcare professionals expressed high acceptance of home spirometry. Parents widely emphasized that home telemonitoring significantly reduces the time costs associated with medical visits. Real-time data transmission facilitated by telemonitorings reshaping intervention models in asthma management, enhancing parental autonomy in managing their children's condition. Additionally, home telemonitoring, as a complementary approach to outpatient treatment, demonstrates unique value in preventing and controlling respiratory infectious diseases. This advantage has drawn significant parental attention post-COVID-19 and has become one of the key drivers for adopting home telemonitoring.so,home spirometry telemonitoring in pediatric patients with asthma is feasible and acceptable,this technology holds potential to improve access to specialized care for asthmatic childrenand alleviate the burden on healthcare systems (23).

Several key obstacles hinder the promotion of home spirometry telemonitoring technologies. These include the absence of a standardized pricing system, exclusion of related equipment from medical insurance, and a lack of legal and regulatory frameworks specifically tailored to telemedicine practices. Currently, there is no domestic or international industry standards for home spirometry telemonitoring. Participant acceptance varied throughout the monitoring process, with 5 participants dropping out within the first week of home monitoring. The main reasons for dropout included travel, perceived inconvenience, irrelevance of monitoring, and absence of symptoms. In contrast to the independence of Western children, Asian schoolchildren rely heavily on parental supervision to manage their asthma. This suggests that researchers must understand the child's family living conditions when designing optimal monitoring programs. Additionally, researchers should provide consistent health education and scientific outreach to both parents and children, improving parental knowledge of disease management and alleviating concerns about data interpretation. In China, pediatricians face challenges such as shortages and heavy workloads. Therefore, administrators should support job creation, better time allocation, and performance incentives when implementing home spirometry telemonitoring.

It has been reported that anxiety, depression, and helplessness negatively impact parents’ ability to effectively manage their child's illness (24). Mandizha J's qualitative survey revealed that participants experienced a wide range of emotions, including worry, uneasiness, indifference, and ambivalence during home spirometry (25). In our interview, uneasiness was the predominant emotion expressed by both the parents and healthcare professionals. This uneasiness among parents predominantly stemmed from an absence of self-assurance in their asthma management knowledge and a concomitant fear that incorrect decisions could exacerbate their child's health condition. Healthcare professionals’ anxiety stemmed from uncertainties regarding the clinical application of this emerging technology. This uneasiness may cause them to adopt a conservative approach in promoting and implementing home spirometry telemonitoring. It is recommended that researchers pay close attention to the psychological burden experienced by participants during home spirometry monitoring. Emotional needs should be fully considered, and efforts should be made to help participants build confidence and alleviate uneasiness through regular interviews, psychological counseling, or by incorporating a counselor into the team.

In the current study, we observed a decreasing trend in adherence to home spirometry in an unsupervised environment. Despite implementing system push notifications, WeChat reminders, and telephone inquiries, we were unable to prevent the gradual decline in adherence. Previous studies have also reported that adherence to home spirometry monitoring tends to decrease over time (26, 27). Additionally, factors such as lack of guidance, feedback, a sense of responsibility, and boredom were identified as major reasons for this decline. It is recommended that future research should providing additional remote support or incorporating fun incentives from medical staff when using unsupervised home spirometry. Furthermore, developing passive collection methods for children's spirometry data may be more effective in improving adherence.

Currently, artificial intelligence (AI) shows great potential in interpreting home spirometry data (28, 29). In interviews, both the families of children and healthcare professionals expressed hope that AI could enhance data interpretation accuracy, enable automatic quality control of operations and data, and build predictive models based on big data to actively provide early warnings of the condition. It was also anticipated that AI algorithms could personalize treatment plans and lifestyle guidance by analyzing the child's historical data. Furthermore, healthcare professionals could proactively reach out to parents and children, thereby reducing the caregiving burden on families.

## 5 Conclusion

This study provides the systematic evaluation of home spirometry telemonitoring (using the PF286 device) for pediatric asthma management in China cultural context. By integrating a mixed-methods design that combines rigorous quantitative validation of device consistency with qualitative insights from semi-structured interviews, we demonstrate the feasibility and reliability of unsupervised home spirometry telemonitoring in Chinese children. Furthermore, we identify unique challenges in technology adoption, such as concerns over data security, health education gaps, and policy limitations, which are pivotal for optimizing localized implementation.

## 6 Limitations

This design presents certain limitations. The absence of a conventional monitoring group may hinder precise evaluation of the clinical advantages of home spirometry telemonitoring compared with standard management. Subsequent research will investigate how monitoring data can trigger clinical interventions, thereby validating the practical value of this technology.

## Data Availability

The original contributions presented in the study are included in the article/Supplementary Material, further inquiries can be directed to the corresponding author.
